# Prevalence and Risk Factors of Anaemia among Children Aged between 6 Months and 14 Years in Kenya

**DOI:** 10.1371/journal.pone.0113756

**Published:** 2014-11-25

**Authors:** Oscar Ngesa, Henry Mwambi

**Affiliations:** School of Mathematics, Statistics and Computer Science, University of KwaZulu-Natal, Pietermaritzburg, KwaZulu-Natal, South Africa; Pennsylvania State University College of Medicine, United States of America

## Abstract

**Background:**

Anaemia is one of the significant public health problems among children in the world. Understanding risk factors of anaemia provides more insight to the nature and types of policies that can be put up to fight anaemia. We estimated the prevalence and risk factors of anaemia in a population-based, cross-sectional survey.

**Methodology:**

Blood samples from 11,711 children aged between 6 months and 14 years were collected using a single-use, spring-loaded, sterile lancet to make a finger prick. Anaemia was measured based on haemoglobin concentration level. The generalized linear model framework was used to analyse the data, in which the response variable was either a child was anemic or not anemic.

**Results:**

The overall prevalence of anaemia among the children in Kenya was estimated to be 28.8%. The risk of anaemia was found to decrease with age progressively with increase in each year of age; children below 1 year were at highest risk of anaemia. The risk of anaemia was significantly higher in male than female children. Mothers with secondary and above education had a protective effect on the risk of anaemia on their children. Malaria diagnosis status of a child was positively associated with risk anaemia.

**Conclusion:**

Controlling co-morbidity of malaria and improving maternal knowledge are potential options for reducing the burden of anaemia.

## Introduction

Anaemia still remains a public health problem in all corners of the world [1]. It is estimated that 293.1 million children under five years are suffering from anaemia and 28.5% of these children are from sub-Saharan Africa [1]. Anaemia aetiology has a multi-factorial nature, but with two main culprits, namely, deficiency in micronutrients and parasitic infections. Malaria is the most common parasitic infection associated with anaemia [2]–[6]. Iron deficiency is the main micronutrient that contributes to anaemia [7].

Half of the burden of childhood anaemia is suspected to be caused by Iron deficiency [1]. In children, the disease is strongly linked with reduced cognitive development, high mortality, low immunity and low growth rate [8], [9]. Anaemia can be life threatening, especially when the haemoglobin concentration level falls below 70g/l [10]. For such severe anaemia cases, common treatment methods are iron supplementation and blood transmission [11]–[15]

In Eastern Africa, it is estimated that three quarters of the children under five suffer from anaemia [16]. Previous studies on anaemia in Kenya have been restricted to particular areas of the country which are malaria endemic [17], [18]. The objective of this paper is to estimate the prevalence of anaemia and to determine its risk factors from a nationwide cross-sectional survey.

## Ethical Statement

This study protocol was approved by the Kenyatta National hospital/University of Nairobi Scientific and Ethics Review Committee. Written informed consent was obtained from the parents or guardians of children who participated in the study. Data were analyzed anonymously. The risks and benefits of participation in the study were explained to each participant during the process of the informed consent. The risk of participation was the discomfort caused by the pricking needle while the benefits included identification and immediate treatment of malaria.

## Study Area

The republic of Kenya sits on 582,646 square kilometers of land and having a coastal strip along the Indian Ocean covering 536 kilometers. Kenya is situated in East Africa between latitude 5° north and 5° south and longitudes 34° east to 42° west. The lowest platform is the sea level and the highest point is at the peak of Mount Kenya, at 5199 meters above the sea level. The changes in altitude and terrain from one region to another, generates disparity in the country's climate, which ranges from hot and humid tropical along the Indian Ocean coast to temperate in the interior parts of the country and extremely dry in the north and northeast parts of the country. Two main seasons of rain are inherent in Kenya; long rains, which occur in the months of April to June and short rains, which occur in the months of October to December. Kenya is divided into 47 independent administrative units called counties.

## Study Design and Selection of Study Participants

The data used in this analysis was derived from the Malaria indicator survey, a nationwide cross-sectional survey with the objective of determining the status of malaria and anaemia among children of the age of 6 months to 14 years old. The survey was conducted between June to August in 2010; malaria transmission is peak in Kenya during this period.

A two-stage cluster sampling with stratification was used in the survey. The first stage involved selection of enumeration areas leading to creation of 1,800 clusters with probability proportional to measure of size, using districts as strata. Out of these 1,800 clusters, 240 clusters were selected and used in the 2010 malaria indicator survey with each cluster having 30 households. In total, 6,538 households, 11,711 children aged 6 months to 14 years, and 5,749 women aged 15–49 were interviewed during the survey. Two questionnaires were used in the survey; the household questionnaire which collected the characteristics of the households and the individual questionnaire which collected information from consenting women aged 15–49 years. Further information on the survey methodology in the 2010 malaria indicator survey can be found in the official report of the survey [19].

Blood samples were collected using a single-use, spring-loaded, sterile lancet to make a finger prick on children aged between 6 months and 14 years whose guardians/parents consented for their children to be tested for anaemia and malaria. On spot testing for malaria was carried out using the CareStart® kit while anaemia was measured using the HemoCue® kit. The tested blood samples were sent for further laboratory smear tests. Malaria diagnostic results reported in this study were from slide microscopy.

## Data Analysis

### Outcome variable

The outcome of interest in this study is anaemia test result. Anaemia status was defined, adjusted for both altitude and age as recommended by World Health Organisation. A child in the age of 6 to 59 months is defined to be anaemic if his/her altitude adjusted haemoglobin is <110 g/L. A child in the age of 5 to 11 years is defined to be anaemic if his/her altitude adjusted haemoglobin is <115 g/L. Finally, a child in the age of 12 to 14 years is defined to be anaemic if his/her altitude adjusted haemoglobin is <120 g/L. Therefore the outcome variable is binary, indicating whether the child is anaemic or not.

### Independent variables

The independent variables consisted of the baseline socio-economic, demographic, and other illness variables that included gender, age, malaria status, residence and wealth quantile. An initial exploratory and univariate analysis was carried out to identify variables to be included in the multivariate analysis. The results of this initial analysis are displayed in Table 1.

**Table 1:** Exploratory data analysis.

		Anemia		
Variable	Category	No	Yes	Total
Age(years)	<1 year	331(60.5)	216(39.5)	547(100)
	1	538(59.2)	371(40.8)	909(100)
	2	721(72.5)	273(27.5)	994(100)
	3	770(79.6)	197(20.4)	967(100)
	4	804(83.2)	162(16.8)	966(100)
	5	519(56.8)	395(43.2)	914(100)
	6	562(63.4)	324(36.6)	886(100)
	7	540(67.3)	262(32.7)	802(100)
	8	589(74.0)	207(26.0)	796(100)
	9	505(75.0)	168(25.0)	673(100)
	10	669(78.5)	183(21.5)	852(100)
	11	494(81.7)	111(18.3)	605(100.0)
	12	449(69.5)	197(30.5)	646(100.0)
	13	455(73.2)	167(26.8)	622(100.0)
	14	396(74.4)	136(25.6)	532(100.0)
Sex of child	Male	4078(70.0)	1750(30.0)	5828(100)
	Female	4264(72.5)	1619(27.5)	5883(100)
Place of residence	Urban	984(77.3)	289(22.7)	1273(100)
	Rural	7358(70.5)	3080(29.5)	10438(100)
Wealth quantile	Richest	1482(78.7)	402(21.3)	1884(100)
	Richer	1583(73.7)	566(26.3)	2149(100)
	Middle	1940(73.1)	715(26.9)	2655(100.0)
	Poorer	1700(69.8)	737(30.2)	2437(100)
	Poorest	1614(63.2)	940(36.8)	2554(100.0)
Sex of head of household	Male	5566(71.4)	2233(28.6)	7799(100.0)
	Female	2776(71.0)	1136(29.0)	3912(100.0)
Mother's highest education	No education	882(64.3)	489(35.7)	1371(100.0)
	Primary incomplete	1576(68.9)	711(31.1)	2287(100.0)
	Primary complete	1177(73.7)	421(26.3)	1598(100.0)
	Secondary incomplete	336(73.2)	123(26.8)	459(100.0)
	Secondary complete	501(79.9)	126(20.1)	627(100.0)
	Higher	197(78.2)	55(21.8)	252(100.0)
Malaria	Yes	640(46.1)	747(53.9)	1387(100)
	No	7436(74.9)	2487(25.1)	9923(100.0)
Usage of Mosquito nets	Yes	5363(70.5)	2240(29.5)	7603(100.0)
	No	2950(72.6)	1116(27.4)	4066(100.0)

### Statistical analysis

The data at hand was analyzed using the generalized linear modeling framework. This is a generalization of the linear regression framework to include response variables that are not necessarily continuous and normally distributed by linking the outcome variable to the independent variables using an appropriate link function. There is a rich literature on generalized linear models which has seen applications in numerous fields of studies [20]. Logistic regression is a member of the generalized linear models and is used for modeling binary data [21]. Maximum likelihood procedure is used in getting the parameter estimates in the generalized linear models framework [22]. The model applied is discussed in detail as follows:

Let 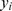 be the anaemia status of child 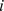. This response variable is defined such that 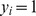 if child 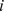 tests positive for anaemia and zero otherwise. The vector 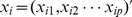 contains 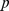 continuous independent random variables and 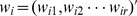 contains 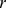 categorical independent random variables with first component accounting for the intercept. This study assumes that the dependent variable 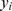 is Bernoulli distributed, i.e 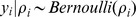 with an unknown mean 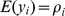, being related to the independent variables as follows:
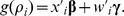
In this equation, 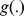 is a logit link function, 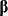 is a 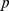 dimensional vector of regression coefficients for the continuous independent variables, and 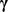 is a 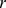 dimensional vector of regression coefficients for the categorical independent variables. The analysis was performed in STATA version 11 (College Station, Texas)

## Results

This study utilized data from 11,711 children across Kenya, in the age of 6 months to 14 years, who provided blood samples for testing. The prevalence of anaemia among children in this age group, in Kenya, was estimated to be 28.8%.

Table 1 shows the prevalence of anaemia by the different demographic, socioeconomic and morbidity factors among the children.

Table 2 provides the parameter estimates of the generalized linear model, employed to determine the risk factors of anaemia among the children of interest in this study. Age of the child, sex of the child, wealth quantile of the household, mother's highest education level and malaria diagnosis status of the child were found to be significantly associated with anaemia while place of residence, sex of the household head and mosquito nets usage, were found to be insignificant. Two way interaction effects between these variables were also included in the model and were found to be insignificant and were dropped from the analysis.

**Table 2:** Estimates of the odds ratio, p-value and corresponding 95% confidence intervals (CI).

	Unadjusted Estimates		Adjusted Estimates	
Variable	P-value	OR(95%CI)	P-value	OR(95%CI)
Age(ref = 14 years)	<0.001	1	<0.001	1
0	<0.001	1.900(1.466,2.463)	<0.001	2.134(1.399,3.257)
1	<0.001	2.008(1.587,2.541)	0.001	2.038(1.356,3.064)
2	0.424	1.103(0.868,1.401)	0.648	1.101(0.729,1.662)
3	0.021	0.745(0.58,0.956)	0.202	0.761(0.499,1.158)
4	<0.001	0.587(0.453,0.759)	0.002	0.514(0.334,0.791)
5	<0.001	2.216(1.753,2.802)	0.001	2.058(1.364,3.107)
6	<0.001	1.679(1.323,2.13)	0.004	1.842(1.214,2.796)
7	0.006	1.413(1.106,1.804)	0.259	1.301(0.824,2.053)
8	0.857	1.023(0.796,1.315)	0.544	0.863(0.537,1.388)
9	0.811	0.969(0.746,1.258)	0.298	0.769(0.469,1.262)
10	0.080	0.796(0.618,1.027)	0.605	0.883(0.55,1.417)
11	0.003	0.654(0.493,0.869)	0.019	0.513(0.294,0.895)
12	0.062	1.278(0.988,1.652)	0.842	1.053(0.632,1.754)
13	0.621	1.069(0.821,1.391)	0.537	0.853(0.514,1.414)
Child's Sex(ref = Female)		1		1
Male	0.003	1.130(1.043,1.224)	0.001	1.215(1.083,1.362)
Place of residence(ref = Urban)		1		1
Rural	<0.001	1.425(1.242,1.636)	0.833	1.022(0.835,1.250)
Wealth quantile(ref = Richest)	<0.001	1	0.001	1
Richer	<0.001	1.318(1.139,1.526)	0.077	1.222(0.979,1.525)
Middle	<0.001	1.359(1.182,1.562)	0.019	1.296(1.044,1.609)
Poorer	<0.001	1.598(1.389,1.839)	0.050	1.249(1.001,1.561)
Poorest	<0.001	2.147(1.873,2.461)	<0.001	1.615(1.284,2.031)
HH head's sex (ref = Female)		1	-	-
Male	0.646	0.980(0.901,1.067)	-	-
Mother's Education(ref = Higher)	<0.001	1	<0.001	1
No education	<0.001	1.986(1.444,2.731)	0.015	1.569(1.090,2.259)
Primary incomplete	0.003	1.616(1.183,2.207)	0.227	1.241(0.874,1.760)
Primary complete	0.128	1.281(0.931,1.763)	0.681	1.077(0.757,1.531)
Secondary incomplete	0.144	1.311(0.912,1.886)	0.855	1.038(0.698,1.542)
Secondary complete	0.566	0.901(0.630,1.287)	0.419	0.854(0.582,1.253)
Malaria status(ref = No)		1		1
Yes	<0.001	3.490(3.111,3.915)	<0.001	4.022(3.399,4.759)
Mosquito nets usage(ref = Yes)		1		1
No	0.020	0.906(0.832,0.986)	0.093	0.881(0.747,1.101)

**Figure 1:**
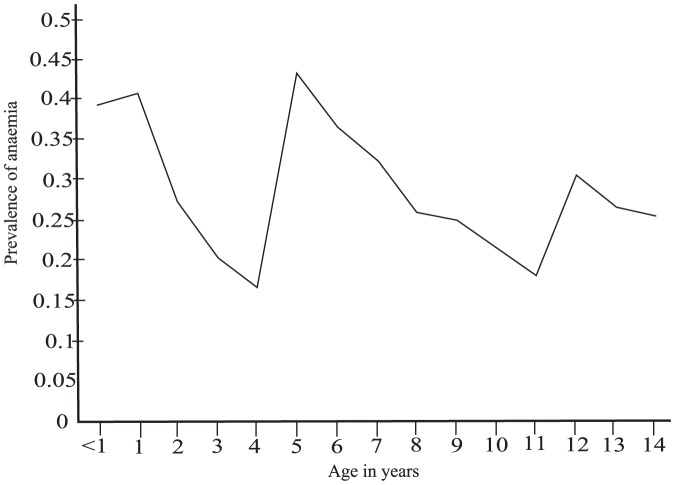
Variation of anaemia prevalence by age.

Age of the child was found to be significantly associated with anaemia (P-value: <0.001). The risk of anaemia was found change with age in a sinusoidal manner. Figure 1 shows the variation of anaemia prevalence by age. A child whose age is less than one year was found to be 2 times more susceptible to anaemia as compared to a 14 year old counterpart (OR: (2.134(1.399, 3.257))).

A male child was found to have a higher risk of presenting anaemia than a female child (OR: 1.215(1.083, 1.362)p-value = 0.001). With regards to wealth quantile, the risk of presenting with anaemia was found to vary in a decreasing manner with highest risk being in the household in the poorest category (OR: 1.615(1.284, 2.031), p-value<0.001). Children in households in the richest quantile had the least risk of anaemia.

Level of mother's education was significantly associated with the risk of anaemia in the children (p-value<0.001). Mothers with post secondary education and secondary education complete had a protective effect on the risk of anaemia on their children. The risk of anaemia was 1.5 times more in children whose mothers had no education as compared to children whose mothers had post secondary education (OR: 1.569(1.09,2.259)). There was a reduction in risk in children whose mothers had completed secondary education; the risk of anaemia was 1.2 times higher in children whose mothers had post secondary education as compared to children whose mothers had completed secondary education only.

Malaria diagnosis status of a child was found to be strongly associated with the risk of anaemia (OR: 4.022(3.399, 4.759), p-value<0.001). Children diagnosed with malaria were found to be 4 times more prone to anaemia.

## Discussion

Anaemia is one of the significant public health problems among children worldwide particularly in malaria prone regions. Understanding risk factors of anaemia provides more insight to the nature and types of policies that can be put up to fight anaemia.

The present study was conducted based on the 2010 Kenya Malaria indicator survey. The study used generalized linear models to identify the risk factors associated with anaemia. This study found the following demographic factors had association with anaemia: age of the child and the child's sex. Malaria diagnosis status was the only morbidity status that was available in the study; it was also found to be strongly related to malaria. The following socio-economic factors were also found to have association with anaemia: mother's education level and wealth quantile of the household. Interaction effects of these variables were all insignificant in the analysis.

From this analysis, it was observed that the risk of having anaemia decreased with age. Children under the age of 1 year were highly susceptible to anaemia. Magalhães [23] carried out an analysis in West Africa, involving Burkina Faso, Ghana and Mali, they reported the same trend of anaemia prevalence by age.

Male children were also found to have an elevated risk of anaemia as compared to their female counterparts. A comparable observation was made in other African countries like Ghana and Malawi [6], [10], [17].

Mother's education level was found to have a protective effect on the chance of the child being diagnosed with anaemia. Children whose mothers' had secondary, and higher levels of education, were less likely to be anaemia positive. Leite et al. [24] had a similar observation on the effect of maternal schooling on anaemia diagnosis among children in their study carried out in Brazil.

Children belonging to households categorized in the poorest quantile have higher chances of testing positive for anaemia. Similar observations were made in previous studies [23], [24]. Significant co-morbidity of anaemia and malaria was found in this study. This is in line with past studies that have reported association in these two morbidities [3]–[6], [17], [25]–[27].

## Conclusions

The results of this study can provide insights to develop policies for intervention of anaemia in a two pronged manner. Firstly is maternal education. Maternal education was found to have a protective effect on risk of anaemia. The government should focus on providing information to young mothers on adequate nutrition for their young babies. Information on food products including indigenous African foods, which contain relevant vitamins and iron, will go a long way in reducing anaemia prevalence in the country [11]–[13], [28], [29].

Secondly, up scaling interventions on malaria and ensuring that these interventions cover most parts of the country will lead to reduction in anaemia cases. This is a consequence of the finding that there is strong association between malaria and anaemia in the children.

Hither to, the study highlights two main areas which could provide avenues for reducing the prevalence of anaemia in Kenya. These areas are, controlling co-morbidity of malaria and anaemia, and improving maternal knowledge.

No study goes without limitations. One major limitation of this study is the cross-sectional nature of the study. Follow-up studies provide more insight into public health problems. Another limitation was due to the fact that anaemia was measured based on hemoglobin concentration only, inclusion of other measures such as C-reactive protein (CRP) and plasma ferriton (PF) concentration [30] could lead to better diagnosis results. Despite these limitations, this present study has determined the prevalence and risk factors of anaemia among children.
