# Correction: Prevalence and Risk Factors of Anaemia among Children Aged between 6 Months and 14 Years in Kenya

**DOI:** 10.1371/journal.pone.0125086

**Published:** 2015-04-08

**Authors:** The PLOS ONE Staff

The second sentence of the Results section of the Abstract is incorrect. The complete, correct sentence is: Across each band of age within which the definition of anaemia remained constant (0–4, 5–11, and 12–14 years old), the prevalence of anaemia declined with each year of age.
